# Facts and Observations Relative to the Influence of Manufactures upon Health and Life

**Published:** 1843-10

**Authors:** 


ART. IV.—*Facts and Observations relative to the Influence of Manufactures upon Health and Life.* By DANIEL NOBLE, Surgeon.—*London*, 1843. 8vo, pp. 88.

THE leading article in our Journal for April last, written at our request by the author, "constitutes (as he states in his preface) the basis of the present publication." Had we not thought the original essay valuable, it would not have found a place in our pages; and we will only say of the work before us, that the great mass of new matter contained in it is of the same important character as that we had the honour of printing. The pamphlet deserves the notice of statisticians and of all inquirers into the etiology of diseases.

